# Supplementary material for: Predictive Risk Factors and Scoring Systems Associated with the Development of Hepatocellular Carcinoma in Chronic Hepatitis B
Source: Cancers (Basel). 2024 Jul 12;16(14):2521. doi: 10.3390/cancers16142521 (PMC11274905; doi:10.3390/cancers16142521)
Supplement: Supplementary file 1 [file cancers-16-02521-s001.zip › cancers-3068765-supplementary.pdf]

Supplementary Table S1. Performance metrics for each prognostic score in the subgroups of HBV patients that received did not receive antiviral or treatment.

TREATED PATIENTS								
	**Cut-off**	**Sensitivity (95% CI)**	**Specificity (95% CI)**	**PPV (95% CI)**	**NPV (95% CI)**	**LR+ (95% CI)**	**LR- (95% CI)**	**Accuracy (95% CI)**
**CU-HCC**	17.25	75.86 % (56.46-89.70)	67.42% (60.00-74.24)	27.50% (22.03-33.74)	94.49% (89.92-97.05)	2.33 (1.73-3.13)	0.36 (0.19-0.69)	68.60% (61.80- 74.86)
**GAG-HCC**	93.6	68% (46.5-85.05)	87.57% (81.63- 92.14)	12.89% (8.52-18.43)	94.87% (91.24-97.05)	5.47 (3.38-8.86)	0.37 (0.21-0.65)	85.05% (79.24-89.75)
**REACH-B**	6.5	94.74% (73.97- 99.87)	25.37% (18.26-33.61)	15.25% (13.47-17.22)	97.14 (83.16-99.57)	1.27 (1.10-1.47)	0.21 (0.03-1.43)	33.99% (26.53-42.07)
**PAGE-B**	16.5	81.48% (61.92-93.70)	73.91% (66.42-80.51)	34.37% (27.63-41.83)	95.97% (91.48-98.14)	3.12 (2.28-4.28)	0.25 (0.11-0.56)	75.00% (68.18-81.02)
**FIB-4**	2.08	74.07% (53.72-88.89)	59.63% (51.62-67.28)	14.36% (9.68-20.20)	93.20% (87.74-96.33)	1.83 (1.37-2.46)	0.43 (0.23-0.83)	61.70% (54.35-68.68)

UNTREATED PATIENTS								
	**Cut-off**	**Sensitivity (95% CI)**	**Specificity (95% CI)**	**PPV (95% CI)**	**NPV (95% CI)**	**LR+ (95% CI)**	**LR- (95% CI)**	**Accuracy (95% CI)**
**CU-HCC**	5	100 % (47.82-100)	64.27% (59.46-68.87)	3.25% (2.87-3.68)	100% (98.63-100)	2.80 (2.46-3.18)	0.00	64.69% (59.92-69.25)
**GAG-HCC**	93.94	100% (39.76-100)	95.15% (92.53-97.06)	17.39% (11.95-24.61)	100% (99.02-100)	20.63 (13.31-31.99)	0.00	95.2% (92.61-97.09)
**REACH-B**	12.5	100% (2.5- 100)	98.16% (95.35-99.50)	20% (8.65-39.76)	100% (98.28-100)	54.25 (20.55-143.24)	0.00	98.17% (93.37-99.50)
**PAGE-B**	15.5	100% (47.82-100)	80.35% (75.76-84.40)	6.85% (5.61-8.34)	100% (98.68-100)	5.09 (4.11-6.30)	0.00	80.63% (76.10-84.63)
**FIB-4**	2.73	100% (47.82-100)	86.80% (86.09-92.79)	12.50% (9.45-16.36)	100% (98.81-100)	9.8 (7.16-13.41)	0.00	89.94% (86.29-92.89)

Abbreviations: GAG-HCC, Guide with Age, Gender, HBV DNA, Core Promoter Mutations and Cirrhosis-HCC; CU-HCC, Chinese University-HCC; REACH-B, risk estimation for hepatocellular carcinoma in chronic hepatitis B; PAGE-B, Platelet Age Gender–HBV; FIB-4, fibrosis-4; PPV, positive predictive value; NPV, negative predictive value; LR, likelihood ratio.

Supplementary Table 2. Performance metrics for each prognostic score in the subgroups of HBV patients with cirrhosis or without cirrhosis.

**PATIENTS WITH CIRRHOSIS**								
	**Cut-off**	**Sensitivity (95% CI)**	**Specificity (95% CI)**	**PPV (95% CI)**	**NPV (95% CI)**	**LR+ (95% CI)**	**LR- (95% CI)**	**Accuracy (95% CI)**
**CU-HCC**	17.25	95.65 % (78.05-99.89)	11.54% (4.35-23.44)	32.35% (29.55-35.29)	85.71% (43.35-97.92)	1.08 (0.95-1.23)	0.38 (0.05-2.95)	37.33% (26.43-49.27)
**GAG-HCC**	103.5	84.2% (60.4-96.6)	40.0% (25.70-55.67)	37.21% (30.34-44.64)	85.71% (66.67-94.74)	1.40 (1.03-1.91)	0.39 (0.13-1.18)	53.12% (40.23-65.72)
**REACH-B**	8.5	91.67% (61.52-99.79)	34.48% (17.94-54.33)	36.67% (29.72-44.22)	90.91 (58.91-98.59)	1.40 (1.02-1.92)	0.24 (0.03-1.69)	51.22% (35.13-67.12)
**PAGE-B**	17	86.96% (66.41-97.22)	40.43% (26.37-55.73)	41.67% (34.97-48.68)	86.36% (67.59-95.06)	1.46 (1.10-1.94)	0.32 (0.11-0.98)	55.71% (43.34-67.59)
**FIB-4**	7.68	39.13% (19.71-61.46)	39.13% (25.09-54.63)	24.32% (15.51-36.01)	56.25% (44.13-67.67)	0.64 (0.37-1.13)	1.56 (0.96-2.53)	39.13% (27.60-51.63)
**PATIENTS WITHOUT CIRRHOSIS**								

	Cut-off	Sensitivity (95% CI)	Specificity (95% CI)	PPV (95% CI)	NPV (95% CI)	LR+ (95% CI)	LR- (95% CI)	Accuracy (95% CI)
**CU-HCC**	1.25	90.91 % (58.72-99.77)	36.45% (32.32-40.73)	2.92% (2.40-3.53)	99.48% (96.71-99.92)	1.43 (1.17-1.74)	0.25 (0.04-1.62)	37.57% (33.45-41.83)
**GAG-HCC**	59.98	100% (69.15-100)	66.13% (61.79-70.28)	5.59% (4.97-6.27)	100% (98.89-100.00)	2.95 (2.61-3.34)	0.00	66.8% (62.52-70.88)
**REACH-B**	6.5	87.5% (47.35- 99.68)	51.91% (46.23-57.56)	4.43% (3.37-5.81)	99.39% (96.29-99.90)	1.82 (1.37-2.42)	0.24 (0.04-1.51)	52.80% (47.18-58.36)
**PAGE-B**	11	100% (66.37-100)	57.84% (53.14-62.43)	4.50% (4.06-4.99)	100% (98.68-100)	2.37 (2.13-2.64)	0.00	58.66% (54.02-63.19)
**FIB-4**	2.21	86.7% (83.21-89.69)	55.56% (21.2-86.3)	7.69% (4.25-13.53)	98.99% (97.92-99.51)	4.18 (2.22-7.84)	0.51 (0.25-1.07)	86.09% (82.58-89.12)

Abbreviations: GAG-HCC, Guide with Age, Gender, HBV DNA, Core Promoter Mutations and Cirrhosis-HCC; CU-HCC, Chinese University-HCC; REACH-B, risk estimation for hepatocellular carcinoma in chronic hepatitis B; PAGE-B, Platelet Age Gender–HBV; FIB-4, fibrosis-4; PPV, positive predictive value; NPV, negative predictive value; LR, likelihood ratio.
